# Read-through transcripts in normal human lung parenchyma are down-regulated in lung adenocarcinoma

**DOI:** 10.18632/oncotarget.8556

**Published:** 2016-04-02

**Authors:** Giulia Pintarelli, Alice Dassano, Chiara E. Cotroneo, Antonella Galvan, Sara Noci, Rocco Piazza, Alessandra Pirola, Roberta Spinelli, Matteo Incarbone, Alessandro Palleschi, Lorenzo Rosso, Luigi Santambrogio, Tommaso A. Dragani, Francesca Colombo

**Affiliations:** ^1^ Department of Predictive and Prevention Medicine, Fondazione IRCCS, Istituto Nazionale dei Tumori, Milan, Italy; ^2^ Department of Health Sciences, University of Milano-Bicocca, Monza, Italy; ^3^ Hematology and Clinical Research Unit, San Gerardo Hospital, Monza, Italy; ^4^ Department of Surgery, San Giuseppe Hospital, Multimedica, Milan, Italy; ^5^ Department of Surgery, IRCCS Fondazione Cà Granda Ospedale Maggiore Policlinico, Università degli Studi di Milano, Milan, Italy; ^6^ Present Address: UCD School of Biomolecular and Biomedical Science, University College Dublin, Belfield, Dublin, Ireland; ^7^ Formerly, Department of Predictive and Prevention Medicine, Fondazione IRCCS, Istituto Nazionale dei Tumori, Milan, Italy; ^8^ Formerly, Department of Health Sciences, University of Milano-Bicocca, Monza, Italy

**Keywords:** conjoined genes, gene fusion, lung adenocarcinoma, RNA-Seq, read-through transcripts

## Abstract

Read-through transcripts result from the continuous transcription of adjacent, similarly oriented genes, with the splicing out of the intergenic region. They have been found in several neoplastic and normal tissues, but their pathophysiological significance is unclear. We used high-throughput sequencing of cDNA fragments (RNA-Seq) to identify read-through transcripts in the non-involved lung tissue of 64 surgically treated lung adenocarcinoma patients. A total of 52 distinct read-through species was identified, with 24 patients having at least one read-through event, up to a maximum of 17 such transcripts in one patient. Sanger sequencing validated 28 of these transcripts and identified an additional 15, for a total of 43 distinct read-through events involving 35 gene pairs. Expression levels of 10 validated read-through transcripts were measured by quantitative PCR in pairs of matched non-involved lung tissue and lung adenocarcinoma tissue from 45 patients. Higher expression levels were observed in normal lung tissue than in the tumor counterpart, with median relative quantification ratios between normal and tumor varying from 1.90 to 7.78; the difference was statistically significant (*P* < 0.001, Wilcoxon's signed-rank test for paired samples) for eight transcripts: *ELAVL1–TIMM44, FAM162B–ZUFSP, IFNAR2–IL10RB, INMT–FAM188B, KIAA1841–C2orf74, NFATC3–PLA2G15, SIRPB1–SIRPD*, and *SHANK3–ACR*. This report documents the presence of read-through transcripts in apparently normal lung tissue, with inter-individual differences in patterns and abundance. It also shows their down-regulation in tumors, suggesting that these chimeric transcripts may function as tumor suppressors in lung tissue.

## INTRODUCTION

Several genetic alterations have been reported to act as driver events in lung tumorigenesis or to modulate the progression of lung tumors and their responses to therapy [1, 2]. Among these alterations, chromosomal rearrangements (e.g. translocations, inversions and insertions) affecting genes encoding receptor tyrosine kinases, such as *ALK*, *ROS1* and *RET*, have been extensively studied [1, 3–9]. These rearrangements can give rise to gene fusions by juxtapositioning previously independent coding sequences; when these fused genes are transcribed, they produce chimeric transcripts.

Chimeric transcripts can also be generated in the absence of chromosomal rearrangements, as a result of the transcriptional “read through” of two adjacent, similarly oriented genes, with the splicing out of the intergenic region [10]. The identification of read-through transcripts has been made easier by the possibility of transcriptome sequencing using next-generation technologies [11]. Through this approach, read-through transcripts have been observed in several tumor types, such as breast, prostate, gastric, and renal cancer [12–18]. Read-through transcripts in cancer tissues may have tumorigenic potential, since their silencing in cancer cell lines reduces cell proliferation [13–15]. In lung cancer, evidence for read-through transcripts is currently limited to our incidental discovery, during a gene expression study, of RNA chimeras formed by the intergenic transcription of the conjoined genes *PPP3R1* and *CNRIP1*, in a few samples of lung adenocarcinoma and normal lung tissue [19].

Even though the presence of read-through transcripts in normal, non-neoplastic tissue is documented [10, 12, 18, 20–23], their physiological role is still unknown. Computational and experimental analyses suggested that chimeric transcripts, including read-throughs, are translated, producing fusion proteins with altered properties, such as new intracellular localizations and new functions, through the novel combination of protein domains [20, 24]. The biological functions of fusion proteins encoded by some read-through transcripts have been primarily investigated in cancer cell lines, in order to understand their role in tumorigenesis: indeed, the effects of read-through transcript silencing on cell proliferation, colony formation, and ability to grow in an anchorage-independent manner (soft agar assay) have been reported [13, 14, 17, 25]. It has also been suggested that untranslated read-through transcripts regulate gene expression at the RNA level, as either non-coding RNA or regulatory RNA [22].

In this study, we used RNA sequencing to search for additional read-through transcripts in normal lung parenchyma from adenocarcinoma patients. Candidate transcripts were examined by PCR followed by Sanger sequencing of the read-through fusion points, permitting the validation of 43 transcripts. Comparison of the expression levels of 10 of these read-through transcripts in paired samples of non-involved lung tissue and lung adenocarcinoma tissue revealed lower levels in tumor tissue than in the normal tissue counterpart.

## RESULTS

### Detection of read-through fusion events

Paired-end whole transcriptome sequencing (RNA-Seq) was carried out on 64 samples of RNA from non-involved lung tissue of 64 lung adenocarcinoma patients (Table 1). The RNA-Seq data were analyzed to identify chimeric transcripts involving two adjacent genes on the same chromosome, in the same orientation. This criterion, together with the fact that the RNA samples were from apparently normal tissue, maximized the probability that the identified chimeric transcripts had been generated by transcriptional read-through events, without genetic aberrations. Among the 64 samples analyzed, 24 had at least one read-through transcript, up to a maximum of 17 different read-through transcripts observed in a single patient. Altogether we found 52 unique read-through species involving 37 different pairs of adjacent genes (Table 2). In some cases, the read-through events involved different exons of the same pair of genes. Most read-through transcripts were found in only one or two patients, but eight were found in 3–10 patients.

**Table 1:** Clinical characteristics of the 64 lung adenocarcinoma patients from whom total RNA from resected non-tumor lung tissue was analyzed by RNA-Seq and of 45 lung adenocarcinoma patients from whom total RNA from non-tumor and tumor lung tissue was analyzed by qPCR (these 45 patients included 6 patients analyzed by RNA-Seq)

	RNA-Seq	qPCR
Sex, *n* (%)		
Men	50 (78.1)	34 (75.6)
Women	14 (21.9)	11 (24.4)
Age at surgery, years, mean (SD)	63.8 (7.3)	62.6 (10.7)
Smoking status, *n*		
Smoker	61	31
Non-smoker	1	11
Missing	2	3
Stage, *n*		
I	55	18
II	3	8
III or IV	5	13
Missing	1	6

**Table 2:** Read-through transcripts identified by RNA-Seq analysis

Read-through name^1^	Chr.^2^	First transcript (exon)^3^	Second transcript (exon)^3^	Intergenic region (bp)	N. patients^4^	PCR validated	Maintained frame
AGRP-ATP6V0D1	16	NM_001138 (3)	NM_004691 (2)	1,334	1	Yes	No
ARF3-FKBP11	12	NM_001659 (4)	NM_001143781 (4)	9,249	1	Different^6^	See Table 3
ARPC4-TTLL3	3	NM_001024959 (5)	NM_001025930 (2)	360	10	Yes	No
CD59-C11orf91	11	NM_203331 (4)	NM_001166692 (2)	NA^5^	2	Different^6^	See Table 3
CHIA-PIFO	1	NM_021797 (8)	NM_181643 (2)	25,722	1	Yes	Yes
CTSC-RAB38	11	NM_001814 (5)	NM_022337 (2)	118,125	2	Yes	Yes
		NM_001814 (4)	NM_022337 (2)		1	No	−
CTSD-IFITM10	11	NM_001909 (8)	NM_001170820 (2)	2,161	3	Yes	Yes
		NM_001909 (7)	NM_001170820 (2)		2	No	−
ELAVL1-TIMM44	19	NM_001419 (4)	NM_006351 (3)	14,658	2	Different^6^	See Table 3
FAM162B-ZUFSP	6	NM_001085480 (3)	NM_145062 (2)	83,406	1	Yes	No
FARSA-SYCE2	19	NM_004461 (12)	NM_001105578 (3)	3,203	1	Yes	Yes
HARS2-ZMAT2	5	NM_012208 (13)	NM_144723 (2)	NA^5^	1	Different^6^	See Table 3
HSD17B11-HSD17B13	4	NM_016245 (5)	NM_178135 (2)	13,704	1	Different^6^	See Table 3
IFNAR2-IL10RB	21	NM_207584 (7)	NM_000628 (2)	683	1	Yes	Yes
		NM_207584 (7)	NM_000628 (3)		1	Yes	No
INMT-FAM188B	7	NM_001199219 (2)	NM_032222 (2)	13,815	4	Yes	No
		NM_001199219 (2)	NM_032222 (3)		7	No	−
		NM_001199219 (3)	NM_032222 (3)		1	No^7^	−
KIAA1841-C2orf74	2	NM_032506 (21)	NM_001143960 (2)	NA^5^	1	Yes	No
LMAN2-MXD3	5	NM_006816 (7)	NM_001142935 (4)	18,805	1	Yes	Yes
MBD1-CFAP53	18	NM_001204139 (14)	NM_145020 (2)	360	1	Yes	No
MED22-SURF6	9	NM_133640 (4)	NM_006753 (2)	1,925	1	Yes	No
MRPS17-GBAS	7	NM_015969 (1)	NM_001202469 (3)	NA^5^	1	No^7^	−
NAA60-CLUAP1	16	NM_001083601 (1)	NM_015041 (2)	13,961	1	Different^6^	See Table 3
NDUFB8-SEC31B	10	NM_005004 (4)	NM_015490 (2)	NA^5^	1	Yes	No
NFATC3-PLA2G15	16	NM_173165 (9)	NM_012320 (2)	16,045	2	Yes	Yes
NKX2-1-SFTA3	14	NM_001079668 (2)	NM_001101341 (4)	2,568	5	Yes	No
		NM_001079668 (2)	NM_001101341 (2)		2	Yes	No
		NM_001079668 (1)	NM_001101341 (2)		1	Yes	No
		NM_001079668 (1)	NM_001101341 (4)		3	Different^6^	See Table 3
		NM_001079668 (1)	NM_001101341 (3)		5	No	−
PACSIN2-ARFGAP3	22	NM_001184970 (10)	NM_001142293 (3)	NA^5^	1	Yes	No
		NM_001184970 (10)	NM_001142293 (4)		1	Different^6^	See Table 3
PIR-FIGF	X	NM_001018109 (9)	NM_004469 (2)	423	1	Yes	No
PLEKHO2-ANKDD1A	15	NM_001195059 (2)	NM_182703 (5)	43,895	1	Yes	Yes
		NM_001195059 (4)	NM_182703 (5)		1	Different^6^	See Table 3
PPRC1-NOLC1	10	NM_015062 (13)	NM_004741 (4)	1,851	1	Different^6^	See Table 3
SCNN1A-TNFRSF1A	12	NM_001159575 (12)	NM_001065 (2)	4,729	2	Yes	Yes
		NM_001159575 (12)	NM_001065 (3)		2	No	−
		NM_001159575 (13)	NM_001065 (2)		1	No^7^	-
		NM_001159575 (10)	NM_001065 (2)		1	No	-
		NM_001159575 (9)	NM_001065 (2)		1	No	-
SFTPC-BMP1	8	NM_003018 (3)	NM_001199 (2)	257	2	Yes	No
SHANK3-ACR	22	NM_033517 (22)	NM_001097 (2)	4,898	1	Yes	Yes
SIRPB1-SIRPD	20	NM_001083910 (2)	NM_178460 (2)	4,678	1	Yes	Yes
SNTB2-VPS4A	16	NM_006750 (6)	NM_013245 (3)	2,304	1	Different^6^	See Table 3
SPECC1L-ADORA2A	22	NM_015330 (13)	NM_000675 (3)	139	1	Different^6^	See Table 3
TSTD1-F11R	1	NM_001113205 (1)	NM_016946 (3)	16,283	2	Different^6^	See Table 3
VAMP8-VAMP5	2	NM_003761 (2)	NM_006634 (2)	2,377	5	Yes	Yes
ZDHHC1-TPPP3	16	NM_013304 (10)	NM_016140 (3)	884	1	Yes	No
ZNF343-SNRPB	20	NM_024325 (6)	NM_198216 (2)	10,964	1	No^7^	-

1 Gene1-Gene2 symbol.

2 Chr.: chromosome.

3 Exons joined by the read-through event.

4 Number of patients in which the read-through transcript was identified by bioinformatic analysis of RNA-Seq data.

5 NA: not available; there is no intergenic distance since genes are overlapping.

6 Identification of different read-through fusion points by Sanger-sequencing (see Table 3).

7 No sequence amplified at PCR

### Validation of the detected read-through fusion events

To confirm the existence of the read-through transcripts identified by RNA-Seq, we carried out PCR, using primers to amplify the sequence spanning the junction, on cDNA from at least one patient in whom each transcript was identified. For four transcripts, PCR did not amplify any sequence so these were excluded from further analysis. For 17 transcripts, we obtained multiple amplicons of different sizes, while for the remaining 31 transcripts we obtained a single amplicon, with 21 of them having the expected molecular weight. Sanger sequencing of the amplicons validated 28 transcripts as predicted by RNA-Seq and excluded seven of them (Table 2). Moreover, for the remaining 13 transcripts, Sanger sequencing did not confirm the predicted fusion point, but instead identified one or two different splicing isoforms involving different exons of the same conjoined genes (Table 3). Hence this analysis validated 28 read-through transcripts among the 52 discovered using RNA-Seq and identified 15 additional read-through transcripts by Sanger sequencing of PCR products, for a total of 43 read-through events involving 35 pairs of genes.

**Table 3:** Read-through transcripts identified by Sanger sequencing of PCR amplicons

Read-through name^1^	First transcript (exon)^2^	Second transcript (exon)^2^	Maintained frame
ARF3-FKBP11	NM_001659 (4)	NM_001143781 (2)	No
	NM_001659 (4)	NM_001143781 (3)	No
CD59-C11orf91	NM_203331 (4)*	NM_001166692 (2)*	No
ELAVL1-TIMM44	NM_001419 (4)	NM_006351 (2)	No
HARS2-ZMAT2	NM_012208 (13)^§^	NM_144723 (2)	No
HSD17B11-HSD17B13	NM_016245 (6)	NM_178135 (2)	No
NAA60-CLUAP1	NM_001083601 (2)	NM_015041 (2)	No
NKX2-1-SFTA3	NM_001079668 (1)	NM_001101341 (2)^§^	No
PACSIN2-ARFGAP3	NM_001184970 (10)	NM_001142293 (2)	No
PLEKHO2-ANKDD1A	NM_001195059 (5)	NM_182703 (4)	Yes
	NM_001195059 (5)	NM_182703 (5)	Yes
PPRC1-NOLC1	NM_015062 (13)	NM_004741 (2)	No
SNTB2-VPS4A	NM_006750 (6)	NM_013245 (2)	Yes
SPECC1L-ADORA2A	NM_015330 (14)	NM_000675 (3)	Yes
TSTD1-F11R	NM_001113205 (1)	NM_016946 (2)	Yes

1 Gene1-Gene2 symbol.

2 Exons joined by the read-through event.

* A portion of intragenic region is included in the read-through transcript.

§ Incomplete exon.

Read-through transcription most frequently involved the second-to-last exon of the first gene, i.e. the one before the 3′-UTR (in 28 of 43 cases, ∼65%), and the second exon of the second gene (in 30 cases, ∼70%). Sometimes, however, the read-through event was more complex. For instance, in the *CD59–C11orf91* transcript, a portion of an intragenic (intronic) region was included. Moreover, in two cases, incomplete exons were found: *HARS2* exon 13 in *HARS2–ZMAT2* and *SEC31B* exon 2 in *NDUFB8–SEC31B*. The median intergenic distance, calculated as the number of base pairs between the end of the first parent gene locus and the start of the second parent gene locus, was 3,941 bp. For 10 gene pairs the intergenic distance was greater than 10 kb, whereas in seven cases it was less than 1 kb. Four validated read-though transcripts originated from overlapping genes. Finally, 17 read-through events maintained the original open reading frames of the two coding sequences that were joined (Tables 2 and 3).

### Expression levels of read-through transcripts in matched samples of non-involved and lung adenocarcinoma tissue

**Figure 1:**
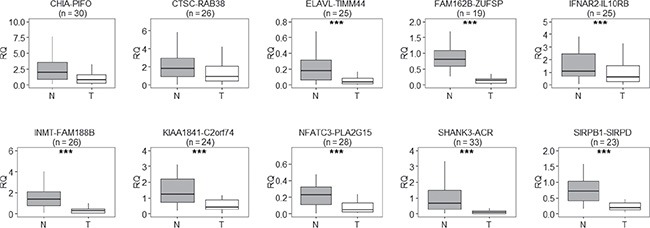
Relative quantification (RQ) of read-through transcripts, in pairs of matched non-involved and tumor tissue from lung adenocarcinoma patients, reveals that read-through transcripts were expressed at lower levels in tumor tissue (T) than in the normal counterpart (N); *n* = number of normal-tumor tissue pairs examined The line within each box represents the median RQ; upper and lower edges of each box are 75^th^ and 25^th^ percentiles, respectively; top and bottom whiskers indicate the greatest and least RQ values, respectively. ***P < 0.001, paired Wilcoxon's signed-rank test for paired samples.

The expression levels of the 43 validated read-through transcripts in non-involved lung tissue were generally low (not shown). To examine their expression levels in lung adenocarcinoma tissue, we focused on 10 transcripts whose levels were among the more abundant and for which only a single amplicon was obtained at PCR in the validation step. These transcripts were: *CHIA–PIFO*, *CTSC–RAB38*, *ELAVL1–TIMM44*, *FAM162B–ZUFSP*, *IFNAR2*(exon 7)*–IL10RB*(exon 2), *INMT–FAM188B*, *KIAA1841–C2orf74*, *NFATC3–PLA2G15*, *SHANK3–ACR*, and *SIRPB1–SIRPD*. We measured, by quantitative PCR, the expression levels of these read-through transcripts in up to 45 pairs of matched tissues of non-tumor lung parenchyma and lung adenocarcinoma tissue. Not all transcripts were measurable in all pairs of samples, and we excluded from further analyses those pairs for which qPCR data were unreliable (i.e. cycle threshold standard deviation > 0.5) in at least one sample. In all cases the median fusion transcript level was higher in normal lung tissue than in the tumor counterpart, and the difference was significant in eight cases (Figure 1). The median relative quantification ratios between normal and tumor varied from 1.90 (*IFNAR2–IL10RB*) to 7.78 (*SHANK3–ACR*). In detail, the difference of expression was statistically significant for *ELAVL1–TIMM44* (*P* = 1.0 × 10^−5^, *n* = 25), *FAM162B–ZUFSP* (*P* = 3.8 × 10^−6^, *n* = 19), *IFNAR2–IL10RB* (*P* = 1.5 × 10^−5^, *n* = 25), *INMT–FAM188B* (*P* = 5.7 × 10^−7^, *n* = 26), *KIAA1841–C2orf74* (*P* = 3.0 × 10^−5^, *n* = 24), *NFATC3–PLA2G15* (*P* = 1.9 × 10^−4^, *n* = 28), *SIRPB1–SIRPD* (*P* = 4.8 × 10^−7^, *n* = 23), and *SHANK3–ACR* (*P* = 1.9 × 10^−7^, *n* = 33; Wilcoxon's signed-rank test for paired samples; Figure 1)

To determine if the expression levels of these 10 read-through transcripts were affected by the patients’ clinical characteristics, we fitted a multivariate linear model, treating sex, smoking habit, and disease stage (stage I or stage > I) as covariates (Table 4). This analysis confirmed that the expression levels were significantly associated with the type of tissue (normal vs. tumor) for the eight fusion transcripts and also found a significant effect for a ninth, namely *CHIA–PIFO* (*P* = 0.0184). Read-through transcript levels were not affected by smoking habit. *FAM162B–ZUFSP* read-through levels were significantly associated with disease stage and sex (*P* = 0.0015 and *P* = 0.019, respectively), whereas those of *NFATC3–PLA2G15* were associated with sex (*P* = 0.040). The expression levels of the other read-through transcripts were not affected by sex or stage.

**Table 4:** Influence of clinical characteristics on read-through transcript levels in lung adenocarcinoma tissue versus non-involved lung tissue, determined by testing in a multivariate linear model, using disease stage, smoking habit and sex as covariates

Read-through^1^	Variable/Covariate^2^	Coefficient estimate	SE^3^	*P*^4^
*CHIA-PIFO*				
	Tissue	−11.88	4.85	0.0184
*ELAVL-TIMM44*				
	Tissue	−13.58	4.44	0.004
*FAM162B-ZUFSP*				
	Tissue	−18.69	1.87	1.45 × 10^−10^
	Sex	−4.982	2.00	0.019
	Stage	6.945	1.96	0.001
*IFNAR2-IL10RB*				
	Tissue	−10.25	4.33	0.024
*INMT-FAM188B*				
	Tissue	−20.33	3.65	2.43 × 10^−6^
*KIAA1841-C2orf74*				
	Tissue	−16.50	3.76	1.22 × 10^−4^
*NFATC3-PLA2G15*				
	Tissue	−15.23	4.40	0.001
	Sex	−11.23	5.28	0.040
*SHANK3-ACR*				
	Tissue	−25.61	3.95	4.27 × 10^−8^
*SIRPB1-SIRPD*				
	Tissue	−18.94	3.76	2.29 × 10^−5^

1 Quantitative levels were rank-transformed to improve the normality distribution of values.

2 Tumor tissue as compared to non-involved tissue; males as compared to females; ever smoker as compared to never smoker; stage > *I* as compared to stage = *I*.

3 SE, standard error.

4 *P* values are reported only if *P* < 0.05; *CTSC-RAB38* showed *P* > 0.05 for all variables.

## DISCUSSION

In this study we searched for read-through transcripts, expressed in the non-involved lung tissue of 64 lung adenocarcinoma patients, by RNA-Seq analysis. We confirmed by Sanger sequencing 43 read-through events, involving 35 pairs of conjoined genes. For 10 of these validated read-through transcripts, quantitative PCR analyses in paired samples of non-involved and tumor tissue revealed that nine were down-regulated in lung adenocarcinoma tissue.

Considering the 35 gene pairs involved in read-through events, 26 had already been reported in the literature [10, 12, 14, 21, 22, 26] or annotated in one or more of the NCBI (http://www.ncbi.nlm.nih.gov/gene/), Ensembl (http://grch37.ensembl.org/index.html), ConjoinG (http://metasystems.riken.jp/conjoing/index), and UCSC (https://genome-euro.ucsc.edu/cgi-bin/hg Gateway?redirect=manual&source=genome.ucsc.edu) databases (Supplementary Table S1). In particular, for 16 gene pairs, the exact read-through fusion point identified in this study had already been described, and for six of them (*INMT–FAM188B*, *MED22–SURF6*, *NDUFB8–SEC31B*, *SCNN1A–TNFRSF1A*, *SIRPB1–SIRPD*, and *SNTB2−VPS4*) the transcripts had already been detected in lung, as indicated by ConjoinG database. For an additional 10 gene pairs, the reported read-through events involved different exons and generated different read-through transcript isoforms. Because of the stringent selection criteria used to select chimeric transcripts (using FusionAnalyser software for RNA-Seq data mining) in our study, it is possible that additional read-through transcripts are present in normal lung tissue and deserve further investigation; indeed, by PCR amplification, besides the candidate read-through transcripts undergoing validation, we detected additional read-through isoforms.

Features of the read-through events identified in this study are comparable with those reported for other read-through transcripts in other tissues. In particular, the median intergenic distance of our read-through fusion events (3,941 bp) is small in comparison to that for all gene pairs in the human genome (estimated as 48 kb [10] and 64 kb [12]); this pattern was already observed in earlier studies on conjoined genes, which reported intergenic distances of 8.5–10 kb [10, 12, 22]. This recurrent finding indicates that read-through events occur more frequently between close genes. Also, the frequent involvement of the exon just before the 3′-UTR of the first parent gene and of the second exon of the second gene in the read-through fusion events has already been observed in other studies of read-through events in different tissues [10, 22].

Almost half of the identified read-through fusion events (∼42%) respected the original open reading frame of the two coding sequences that were joined. This percentage is slightly higher than the 25% observed by Akiva et al. [10]. When the reading frame is maintained, it is possible that a bifunctional fusion protein, containing domains of both the original proteins, can be produced, as already demonstrated for TWE–PRIL and Kua–UEV1 proteins [27, 28]. In other cases, when the read-through event causes a frame shift, new proteins may be created with an intact part of the first parent protein fused to an alternative isoform of the second. Even when read-through transcripts are not translated into protein, they may have a role in regulating the expression of the parent genes [10]. Further studies are needed to clarify whether the read-through transcripts identified here are translated into proteins or exert a regulatory function in normal lung tissue.

Until now, read-through transcripts were typically found to be expressed at higher levels in tumor tissue of different cancer types than in the normal tissue counterpart [13, 14, 17, 25], leading to the suggestion that read-through events were involved in carcinogenesis and tumor cell growth. Here, we show that chimeric transcripts, produced by the transcriptional read-through of two adjacent genes, are frequently present in normal lung tissue; a similar phenomenon has recently been observed in human prostate cells [18]. Interestingly, nine of the read-through transcripts identified here were significantly down-regulated in lung adenocarcinoma tissue.

Multivariate statistical modeling did not find any influence of smoking habit on the expression levels of the read-through transcripts. Therefore, it is unlikely that these read-through transcripts originate from some alteration in transcription caused in lung tissue by tobacco smoking. Also sex and tumor stage did not influence read-through transcript levels, except for *FAM162B–ZUFSP* which had a significant association with both covariates and for *NFATC3–PLA2G15* whose levels were associated with sex. These results suggest that sex and stage may play only a minor role, if any, in the mechanisms behind our finding of a down-regulation of these read-through transcript in lung adenocarcinoma tissue. Therefore, after excluding smoking habit, sex and stage as confounders, we speculate that the down-regulation of these read-through transcripts in tumor tissue may be involved in the pathogenetic mechanism of lung tumorigenesis.

For five of the nine significantly down-regulated read-through transcripts (*CHIA–PIFO*, *IFNAR2–IL10RB*, *NFATC3*–*PLA2G15*, *SIRPB1*–*SIRPD*, and *SHANK3*–*ACR*), the reading frames of the two parent genes were maintained, suggesting that a chimeric protein could be produced. It will be interesting to investigate the existence of such proteins in normal lung tissue and in tumor tissue, and to determine if they have any functional role in lung adenocarcinoma pathogenesis. For the other read-through transcripts, where the reading frame was not conserved and a premature stop codon was introduced, it will be interesting to investigate a possible regulatory role in the expression of the protein encoded by the first parent gene. Indeed, it has been suggested that these kinds of read-through events suppress the expression of the upstream gene through the nonsense-mediated decay mechanism [10, 29].

In summary, this report documents the existence of read-through transcripts in normal lung parenchyma that are down-regulated in tumor tissues of adenocarcinoma patients. The possible functions of these read-through transcripts and their role in lung tumorigenesis remain to be elucidated.

## MATERIALS AND METHODS

### Tissue and RNA samples

This study used tissue samples held in a biobank at Fondazione IRCCS Istituto Nazionale dei Tumori, Milan, Italy. The samples consisted of lung adenocarcinoma and matched non-involved (apparently normal) lung parenchyma excised from patients who underwent lobectomy for lung adenocarcinoma in the authors’ institutes. Methods for the collection of samples and associated clinical data have already been reported [19]. Collection of tissue samples and clinicopathological information from patients had been undertaken with ethical review board approval and informed consent in accordance with the tenets of the Declaration of Helsinki. Specifically, the study protocol was approved by the Committees for Ethics of the institutes involved in recruitment (Fondazione IRCCS Istituto Nazionale dei Tumori, San Giuseppe Hospital, IRCCS Fondazione Cà Granda Ospedale Maggiore Policlinico).

For this study, we used RNA from 103 samples of non-involved lung tissue (64 for RNA-Seq and 45, including 6 of those used for RNA-Seq, for quantitative PCR) and 45 samples of lung adenocarcinoma. The RNA had been extracted for previous studies [19, 30–32]. The clinical characteristics of the corresponding 103 patients are reported in Table 1.

### RNA-Seq and identification of read-through transcripts

RNA-Seq was carried out for 64 samples of non-involved lung tissue as previously described [19]. Qseq files, containing the raw sequencing data, were de-indexed and converted to the Sanger FastQ file format. FastQ sequences were aligned to the human genome assembly (GRCh37/hg19 version) using TopHat v.1.2.0 software [33]. The resulting Sequence Alignment/Map (SAM) files were quality-tested using SAM-Profiler software [34], and only those with high mean read quality (> 30 Phred) were analyzed with FusionAnalyser software [35]. Candidate chimeric events were considered all those detected by ≥ 10 independent reads with a mean read quality ≥ 25 and with at least 1 read mapping across the predicted exonic breakpoint. These events were further filtered according to their reciprocal mapping and strandness: only adjacent genes mapping to the same gDNA strand were considered as correct read-through predictions and subsequently tested by Sanger sequencing.

### Amplification and Sanger sequencing

cDNA was synthesized, from the 64 RNA samples (1 μg each) analyzed by RNA-Seq, using the SuperScript VILO cDNA Synthesis Kit (Life Technologies, Foster City, CA, USA). To verify the existence of the identified read-through transcripts in non-involved lung tissue, we amplified the junction regions, using cDNA from at least one sample in which the transcripts were first identified by RNA-Seq. PCR reactions were performed using 40 ng cDNA, 0.6 U AmpliTaq Gold DNA Polymerase (Life Technologies), 0.2 μM primers spanning the junction between transcripts (sequences available upon request), 1.5 mM MgCl_2_, 0.1 mM dNTPs, in a final volume of 25 μl. Amplified fragments were visualized on 4% agarose gels together with Φ × 174 DNA-Hae III digest used as DNA molecular weight marker. Amplicons were Sanger-sequenced at Eurofins MWG Operon (Ebersberg, Germany), to verify the sequence at the transcript fusion site.

### Quantitative PCR

RNA (1 μg) from 45 pairs of non-involved lung tissue and corresponding lung adenocarcinoma tissue was used to synthesize cDNA by reverse transcription using the Transcriptor First Strand cDNA Synthesis Kit (Roche, Basel, Switzerland). To measure expression of the read-through transcripts in tissue pairs, we did quantitative PCR (qPCR) assays with 12.5 ng cDNA template diluted in RNase-free water, 5 μl 2× Fast SYBR Green Master Mix (Life Technologies), and 0.3 μM primers (sequences available upon request; these primers differed from those used to validate read-through fusion points, since qPCR amplicons must be relatively short, i.e. 70–100 bp, whereas PCR amplicons for Sanger sequencing need to be longer) in a final volume of 10 μl. The human hypoxanthine phosphoribosyltransferase 1 (*HPRT1*) gene was used to normalize expression data. Reactions were run in duplicate using the QuantStudio 12K Flex or 7900 HT Fast real-time PCR system (Life Technologies). We considered as unreliable, and thus excluded, qPCR data of those pairs in which either the non-involved or tumor sample, or both, had a cycle threshold (Ct) standard deviation > 0.5. For this reason, a different number of analyzed pairs is reported for each read-through transcript in Figure 1. RQ values for each read-through transcript were calculated, with ExpressionSuite Software v1.0.4 (Life Technologies), with respect to the RQ of the same transcript measured in a calibrator sample, composed of a pool of cDNAs from normal lung tissue samples previously found by RNA-Seq to be positive for the measured read-through transcripts.

### Statistical analyses

Differences in transcript expression levels between normal and tumor tissues were tested for significance using the non-parametric Wilcoxon's signed-rank test for paired samples. The effects of clinical characteristics on transcript levels were tested for significance in a multivariate linear model on rank-transformed data [36]. Both statistical tests were executed using the R graphical user interface “R Commander” [37].

## SUPPLEMENTARY MATERIALS TABLES


