# Supplementary material for: Read-through transcripts in normal human lung parenchyma are down-regulated in lung adenocarcinoma
Source: Oncotarget. 2016 Apr 2;7(19):27889–98. doi: 10.18632/oncotarget.8556 (PMC5053695; doi:10.18632/oncotarget.8556)
Supplement: Supplementary file 1 [file oncotarget-07-27889-s001.pdf]

Prospective validation of microRNA signatures for detecting pancreatic malignant transformation in endoscopic-ultrasound guided fine-needle aspiration biopsies

Supplementary Materials

Supplementary Table S1: Areas under the ROC curve for assessing combinations of miRNAs for discriminating between malignant and non-malignant pancreatic EUS-FNAs

	miR-21	miR-10b	miR-155	miR-196a	miR-210	miR-135b/24
**miR-21**		0.841 (0.731–0.952)	**0.930 (0.868–0.992)**	0.873 (0.778–0.968)	0.852 (0.752–0.952)	0.837 (0.729–0.945)
**miR-10b**			0.926 (0.858–0.994)	0.837 (0.728–0.947)	0.831 (0.716–0.945)	0.820 (0.704–0.936)
**miR-155**				0.873 (0.778–0.968)	0.872 (0.775–0.968)	0.877 (0.782–0.972)
**miR-196a**					0.778 (0.658–0.898)	0.772 (0.648–0.897)
**miR-210**						0.790 (0.672–0.907)
**miR-135b/24**						

AUC (95% CI).

Supplementary Table S2: Demographic and clinical description of validation EUS-FNA and FFPE cohorts

	Validation EUS-FNAs			Validation FFPE		
	**Benign/ Inflammatory/ Pseudocyst (*n* = 3)**	**PDAC (*n* = 7)**	***P***	**Benign (IPMN, *n* = 5)**	**Malignant (CEI, *n* = 5; PDAC (*n* = 6)**	***P***
Age (years)	50.4 ± 26.7	70.3 ± 9.3	0.102†	64.0 ± 9.7	71.8 ± 7.5	0.095†
Female gender (%)	2 (66.7%)	3 (42.9%)	0.490☼	4 (80%)	3 (27.3%)	**0.049**☼
Symptoms	3 (100%)	7 (100%)	NS☼	3 (60%)	8 (72.2%)	0.611☼
Size (mm)	22.3 ± 7.5	28.6 ± 7.5	0.262†	24 ± 9.1	32.8 ± 8.5	0.074†
Solid or cystic	1/2	7/0	**0.016**☼	1/4	9/2	**0.018**☼
No. of fine needle passes	1.7 ± 0.6	3.0 ± 0.6	**0.010**†	1.6 ± 0.5	2.3 ± 0.6	0.061†
RNA yield (ng/μl)	274.1 ± 210.4	59.8 ± 70.3	**0.033**†	258.7 ± 179.0	248.1 ± 135.9	0.895†

PDAC, Pancreatic Ductal Adenocarcinoma; IPMN, intraductal papillary mucinous neoplasm; CEI, Carcinoma-ex-IPMN. ☼Chi-square test. †Unpaired *t*-test. NS, non-significant.
